# Differing Roles of the Face and Voice in Early Human Communication: Roots of Language in Multimodal Expression

**DOI:** 10.3389/fcomm.2017.00010

**Published:** 2017-09-15

**Authors:** Yuna Jhang, Beau Franklin, Heather L. Ramsdell-Hudock, D. Kimbrough Oller

**Affiliations:** 1 Department of Speech Language Pathology and Audiology, Chung Shan University, Taichung, Taiwan; 2 The Institute for Research and Rehabilitation, Memorial Hermann Healthcare, Houston, TX, United States; 3 Department of Communication Sciences and Disorders, Idaho State University, Pocatello, ID, United States; 4 School of Communication Sciences and Disorders, The University of Memphis, Memphis, TN, United States; 5 Konrad Lorenz Institute for Evolution and Cognition Research, Klosterneuburg, Austria; 6 Institute for Intelligent Systems, The University of Memphis, Memphis, TN, United States

**Keywords:** infant vocalization, facial affect, vocal affect, multimodal communication, communication

## Abstract

Seeking roots of language, we probed infant facial expressions and vocalizations. Both have roles in language, but the voice plays an especially flexible role, expressing a variety of functions and affect conditions with the same vocal categories—a word can be produced with many different affective flavors. This requirement of language is seen in very early infant vocalizations. We examined the extent to which affect is transmitted by early vocal categories termed “protophones” (squeals, vowel-like sounds, and growls) and by their co-occurring facial expressions, and similarly the extent to which vocal type is transmitted by the voice and co-occurring facial expressions. Our coder agreement data suggest infant affect during protophones was most reliably transmitted by the face (judged in video-only), while vocal type was transmitted most reliably by the voice (judged in audio-only). Voice alone transmitted negative affect more reliably than neutral or positive affect, suggesting infant protophones may be used especially to call for attention when the infant is in distress. By contrast, the face alone provided no significant information about protophone categories. Indeed coders in VID could scarcely recognize the difference between silence and voice when coding protophones in VID. The results suggest that partial decoupling of communicative roles for face and voice occurs even in the first months of life. Affect in infancy appears to be transmitted in a way that audio and video aspects are flexibly interwoven, as in mature language.

## INTRODUCTION

The goal of the article is to contrast the roles of the face and the voice in affect expression and in infant vocalization types in the first year of human life. Differentiation of these roles is essential in illuminating the origins of spoken language, where the face predominantly expresses affect, while the voice is used also to form words that express referential meanings, name objects, and provide a basis for sentences. Even in the first year, this facial/vocal differentiation can be seen, as manifest in our coder agreement data about affect expression and vocal type.

### Decoupling of Vocal and Facial Actions in Human Language and Infant Vocalization

The voice has a privileged role in language, a role requiring flexible expression of emotional state with all vocal categories, including all words, phrases, and sentences in natural languages. Consequently, we reason that the study of coder agreement regarding infant affect and vocal type transmitted through both face and voice may help reveal foundations for flexible transmission of differing communicative functions at all levels of linguistic expression.

The massive numbers of words in any natural language consist of learned associations between syllables or syllable sequences and references (meanings). In important ways, learned associations for words in language are arbitrary (de Saussure, 1968)—a rose by any other name would still have the color and smell of a rose. The word “rose” obligatorily invokes the idea of roses and can on any occasion be used to perform a variety of illocutionary functions.^1^ Thus, we can name a rose, request a rose, offer a rose, and so on, all by using the word “rose.” These are fundamental features of vocabulary in language that make it open-ended, allowing language to invoke concepts from the present, the past, or the future, and allowing words to be adapted to immediate illocutionary intents in each act of communication. Of particular importance to the present work, it is possible for humans to produce any word while simultaneously producing a wide variety of facial expressions, denoting different conditions of affect, and the differing affect on differing occasions can help specify how, for example, the word “rose” can be used to request, offer, etc.

Our study focuses on three phonatory categories corresponding to protophone types (Section S1A in Supplementary Material for definitions of precanonical and canonical protophones): Vowel-like sounds (hereafter vocants), squeals, and growls, the presumed precursors to words and syllables of language. Even in the first 3 months, these protophones can be associated with functions flexibly. This implies the protophones are not bound to particular expressions of affect, but vary from occasion to occasion, sometimes expressing negativity (complaint), sometimes positivity (exultation), and on other occasions no obvious affect (Scheiner et al., 2006; Oller et al., 2013; Iyer and Ertmer, 2014). This flexibility of infant vocalization continues in the second-half year in canonical babbling (e.g., [baba] or [dada]) (Oller, 1980; Stark, 1980), where infants can also express a variety of affective states while they produce the phonatory features of squeals, vocants, and growls along with the supraglottal articulations of canonical syllables.

Cry and laughter are not so adaptable as syllables or words. They are bound to transmission in and about the here-and-now and are much more consistently associated with particular illocutionary functions (respectively, distress expression and delight/affiliation expression) and corresponding affective states than syllables or words are. This association is reflected in facial displays of negativity for cry and positivity for laughter. In early infancy, cry and laughter are even more tightly bound to their expected affect types than later in life (Sroufe and Wunsch, 1972; Stark and Nathanson, 1974; Green et al., 1987).

A prior article from our laboratory (Oller et al., 2013) pointed out that functional flexibility as seen in human infant protophones has not yet been reported in any non-human primate.^2^ The findings support our observations above by showing that the three most salient protophones (squeals, vocants, growls) were not only produced at high rates compared to cry and laugh but were produced even as young as three months in flexible relation with facial affect types (positive, neutral, negative).^3^ These flexible relations were confirmed by high effect sizes and odds ratios. Although the flexible use of infant protophones was confirmed, the roles face and voice play independently and conjointly in early communication were not investigated.

In contrast to the voice, human facial expressions transmit particular conditions of affect with much greater consistency throughout life (Sroufe, 1995). From birth, infants show negative facial affect e.g., during crying, and by the fourth week, wakeful infants display positive facial affect in response to external stimuli (e.g., presentation of mother voice or of a human face, cf. Sroufe and Waters, 1976). Thereafter, smiling remains positive and frowning negative throughout the first year. In the present study, all the infants were at least 3 months old, and therefore, we assume the infant faces we coded could portray positivity, negativity, and neutrality of affect expression.

Both in infancy and later in life, the face and voice play distinguishable roles in communication. Facial expression does not show decoupling from affect and accompanying illocutionary function to the extent that human vocal categories do. Infants smiling or frowning are seen as expressing positive or negative affect, respectively. Yet no protophone has such a regular pairing with any affect condition.

Our research focuses on infant affect transmission during vocalization because affect naturally constrains the range of illocutionary and perlocutionary forces (see text footnote ^1^) in infant vocal communication to certain valence classes (positive, neutral, or negative, Section S1B in Supplementary Material on affect and communicative function). Positive affect during vocalization can be interpreted by caregiver/receivers as exultation, encouragement to continue interaction, and so on, all of which are naturally positive illocutions (Oller et al., 2013). By contrast, negative affect can be interpreted as rejection, complaint, or mere distress expression, all of which are naturally negative illocutions. In accord with the valence constraint, positive illocutions are constrained to remain within their valence class by their affect, and thus, positive affect during an infant vocalization cannot, for example, be interpreted as complaint. Thus affect transmission (even transmission of neutral affect) is a key factor in determining the functions of communicative acts.

While we know that each protophone type can be accompanied by varying facial affect, we do not know the extent to which protophones may *transmit* affect independent of facial expression. Further, we do not know the extent to which whatever affect the voice may transmit is concordant with that transmitted by the face. Therefore, we address questions about flexible functions in vocal communication by exploring ways affect is transmitted by face and voice individually and jointly, and ways particular combinations of face and voice afford freedom to vocal categories to express affect flexibly.

### A Closer Look at Vocal and Facial Communication

It has recently been argued that the default mode for vocal communication in all primates, including humans, is multisensory/multimodal, with face and voice routinely involved at all levels of individual behavioral events—from production of utterances, to perception by conspecifics, to brain activation of both sender and receiver integrated across a variety of brain regions (Rosenblum, 2005; Ghazanfar, 2010). Non-human primates react more quickly and more accurately to coherent multimodal expressions than to unimodal ones (Chandrasekaran et al., 2011). It has been argued that the integration across modalities “is ubiquitous and automatic” not just in non-human primates but also among humans and “is similar across all individuals across all cultures. The two modalities seem to be integrated even at the earliest stages of human cognitive development” (Ghazanfar, 2013, p. 1441).

Indeed human facial and vocal expressions have been empirically verified to co-occur from as early as the first 3 months of life (Yale et al., 1999; Delgado et al., 2002). The degree of coordination across modalities has been shown to influence patterns of vocabulary growth in the second year (Parladé and Iverson, 2011). Research on non-human primates has been interpreted as suggesting that co-occurrence of vocal production and facial movement may be obligatory in primates generally (Ghazanfar and Logothetis, 2003). In human speech, such co-occurrence is also common; the face must move during most vocalization, because speech overwhelmingly consists of sequences of syllables that must be articulated with movements of the supraglottal tract, that is, the lips, the tongue, and the jaw. Nevertheless, some human vocalizations can be produced with essentially no facial movement—we can close our mouths and say “mmmm,” and we can do this with a variety of different facial expressions. An observer with only visual information would not be able to tell whether vocalization occurs in such cases because the sound is entirely glottal in origin. Thus, while we normally use face and voice together in speech, we have the capacity to produce at least some vocal categories entirely independently of any facial movement.

In addition, human infants seem to show flexibility not just in whether facial and vocal actions co-occur, but also (as indicated above) in how facial and vocal types are associated when they do co-occur (Oller et al., 2013). While it is assumed that this flexibility of vocal action is universal in infancy, there has actually been no cross-cultural research to confirm the assumption. In the present study, we examine intercoder agreement on judgments of facial affect and vocal type with audio-only (AU), video-only (VID), and audio-video (AV) and the extent to which the voice and co-occurring facial expression transmit affect concordantly. These issues have not been investigated previously with regard to the protophones. It is not even clear to what extent the infant voice (through protophone production) is capable of transmitting affect information at all.

### Human Affect Judged in Different Modalities

In adult human communication, it has been shown that prosodic aspects of speech transmit considerable information about affect, independent of facial expression. Recent work, for example, addressed adult judgments based on AU, VID, and AV presentation from recordings of adult actors portraying various affect conditions while pronouncing nonsense sentences (Bänziger et al., 2009). The findings suggest that across several conditions, observers were considerably more accurate in judging affect with VID than AU, and in general only slightly better with AV than with VID. This pattern of results is consistent with the idea that human facial expression is specifically adapted for affect transmission, while the voice may be more weakly associated with affect in humans. A detachment of at least one modality of communication from obligatory affect transmission would seem to be an absolute requirement for language, as argued above. Further, the results from the cited study (Bänziger et al., 2009) suggested that AU provided more reliable information about negative affect than about positive or neutral affect, and in some cases was as good as or better than VID in transmitting negative affect (i.e., anxiety and hot anger in the adult study). This result suggests the possibility that the vocal modality in humans is adapted especially for transmission of negative affect, facilitating communication by senders in distress toward receivers not in visual contact. Thus for cases of conflict or danger (as in aggression and warning) where obtaining visual attention is important, the voice can effectively transmit negativity and urgency. In spite of its special utility in transmitting negativity, if we take account of how the voice is used in language, it is clearly free to be adapted to any of a wide variety of illocutionary or semantic purposes, regardless of intended affective valence. The present work will offer perspective on these findings and speculations about the origins of language within the first year of human life by evaluating the transmission of affect through AU, VID, and AV.

The work will also consider the possibility that facial configurations may play an independent role in the transmission/interpretation of vocal type in human infants. The proposition that the face may show obligatory configurations in combination with particular vocal types (Ghazanfar and Logothetis, 2003) is of special interest here. In humans, vocal communication seems to be founded on a principle of strong detachment of the voice from particular affect requirements (except in cases such as cry and laugh). But in other primates the extent of such detachment appears to be more limited and is, as indicated above, a matter of ongoing investigation. In the human infant, particular affect conditions are not obligatorily associated with particular protophones, and further it seems possible that some protophones can be produced with virtually no facial actions—we propose to evaluate whether it is possible even to recognize the occurrence of early protophones in the absence of audio.

### Strategies for the Present Work

In the prior work (Oller et al., 2013), vocal type (i.e., squeal, growl, vocant, cry, and laugh) was categorized by coders with AU and facial affect with VID. In the present work, multiple observers coded recordings in three separate passes (AU, VID, and AV) for both affect and vocal type. A subset of this design has been applied previously by Green et al. (1995), who studied infant cry and non-cry sounds judged in AU and AV. Our effort included coding of cry and laughter, but the primary intent was to address the roots of language by evaluating the protophones, and all the coder agreement comparisons reported below concern protophones only.

We reason that intercoder agreement provides the best available measure of reliability of transmission for infant vocalizations and infant affect (Section S1C in Supplementary Material for justification of this conclusion). Higher agreement on, for example, VID judgments of infant affect than AU judgments would suggest that the face transmits affect more reliably than the voice. Similarly, higher agreement on judgments of negativity than on positivity would suggest that positivity is less reliably transmitted than negativity. Assessing agreement across coders (none of whom is treated as more valued than another) is required because there is no obvious gold-standard such as that in the previously cited adult work (Bänziger et al., 2009), where actors provided gold-standard stimuli, having been instructed to produce each utterance with a particular type of affect. We cannot be sure of infant state/intent and thus must use coder judgment as a proxy for it.

### Hypotheses

#### Affect Hypotheses, Agreement across Modalities

1 The infant voice transmits affect in protophones, but most effectively for negative affect: the hypothesis predicts statistically reliable intercoder agreement for affect in the AU condition (AU), highest agreement for negativity.
2 The infant face transmits affect more reliably than the voice during protophones: the hypothesis predicts intercoder agreement for affect judged in VID to be statistically reliably higher than in AU. It is anticipated that Hypothesis 2 will be confirmed for all three affect types, with very large effect sizes for positivity and neutrality, and a smaller effect size for negativity.
3 The infant voice and face together transmit affect most reliably: the hypothesis predicts intercoder agreement for affect judged in AV to be statistically reliably higher than in VID or AU.

#### Affect Hypotheses, Concordance of AU, and VID Judgments

4 Infant affect judgments will be concordant across AU and VID: the hypothesis predicts that disagreements between AU and VID judgments of affect will be rare (<10%).
5 The face will predominate in transmission of infant affect: the hypothesis predicts that for conflicting judgments across AU and VID, AV will agree most with VID.

#### Vocal Type Hypotheses, Agreement across Modalities

6 Infant vocal types (squeal, vocant, growl) will be transmitted significantly better than chance by the face alone: the hypothesis predicts intercoder agreement will be statistically reliable in VID (assuming there may be a lipreading component in protophone identification).
7 Infant vocal types will be transmitted better by voice than by face: the hypothesis predicts intercoder agreement of vocal type judgment in AU to be better than in VID.
8 Infant vocal types will be transmitted better by a combination of face and voice than by voice alone: the hypothesis predicts intercoder agreement in AV to be better than in AU or VID.

#### Vocal Type Hypothesis, Detection of Protophones by VID

9 Infant protophones will be differentiable from silence with facial cues only: the hypothesis predicts better than chance agreement in detecting silence as opposed to protophones in VID (assuming there may be a lipreading component in noticing the occurrence of protophones).

## METHODOLOGY

### Source of Recordings for the Present Study

The recordings for the present study are a subset of those used in the prior study (Oller et al., 2013), with number of recordings (9 of the 54 from the prior study) we used being determined by the amount of coding time it was possible to allocate. For each selected recording, the present study required 27 separate new passes of coding for each coder (i.e., 9 infant sessions, each coded in three ways: AU, VID, and AV by each coder), and the 27 passes were required in both affect and vocal type coding.

**FIGURE 1:**
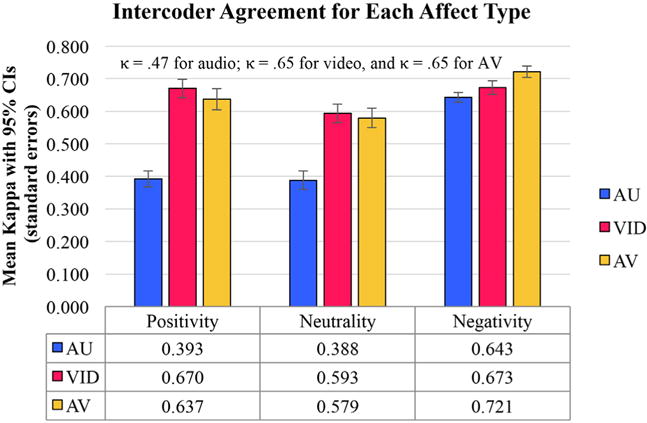
The data indicate that audio-only (AU) (blue bar in the right-hand cluster) was about as effective in transmitting negativity as video-only (VID) (red bar in the right-hand cluster), but that AU was considerably less effective in transmitting positivity or neutrality than the other conditions (blue bars in the middle and left-hand clusters). VID (red bars in the middle and left-hand clusters) was significantly more reliable than AU for positivity and neutrality. Only for negativity did the audio-video (AV) condition (yellow bar, right-hand cluster) yield highest agreement. 95% confidence intervals are included. Kappas at the top of the figure are means.

The prior study was longitudinal, involving recordings from nine infants at each of three ages. In that study, the authors anticipated changes with age on key parameters, and thus the study evaluated each infant at each age. In contrast, the present study is directed at coder agreement with regard to face and voice judgments. While coder agreement across age could in principle differ, an analysis based on a breakdown to three age groups showed that the basic data pattern of Figure 1 applied to all the ages.^4^ Our goal has been to sample from the whole first year and to include samples from all the nine infants available. Therefore, we selected one recording from each of the nine infants (see Table 1). This approach represented a compromise to obtain data from different ages and different infants, while offering, we assumed, good power to evaluate the questions of coder agreement—the data below bear out our power assumption, since the results show many large and highly significant effects.

### Infants and Recordings

A written consent form and a simple questionnaire were completed by infants’ parents before any recordings for the study (Oller et al., 2013). Inclusion criteria were no language, hearing, or developmental disorders. All procedures were approved by The University of Memphis Institutional Review Board for the Protection of Human Subjects.

The infants were not selected to represent any particular language backgrounds, but rather on the basis of the inclusion criteria and the willingness of parents to commit to the longitudinal study. Two of the families turned out to have significant amounts of languages other than English in the homes. For one infant the language was Ukranian, and for the other it was a combination of German and Spanish. All the nine infants were somewhat vocally differentiable (Oller et al., 2013), but all also used all three protophone types, all three facial affect types, and all showed functional flexibility in their combinations of facial affect and protophone usage. We found no reason to conclude that the individual infants were differentiable on protophone usage or facial affect *because* of differences in language(s) in the home. However, we acknowledge that our study pertains to infants growing up in homes in the US, and there remains the possibility of some cross-cultural variation with regard to patterns reported here, both in terms of how infants quantitatively express themselves and how observers judge those expressions.

### Selection of Coding Samples and Utterances

Each of the nine sessions represented in Table 1 were required to contain at least 75 vocalizations (cries and laughs included), as indicated by coding from the prior study. The nine sessions contained a mixture of parent–infant naturalistic interactions and infant play occurring while the research staff interviewed the parents.^5^

### Coding

The coding and boundary placement for each utterance evaluated within each recording was conducted within a software environment (Action Analysis Coding and Training software, AACT) (Delgado et al., 2010) that coordinates frame accurate video and audio presentation with real-time acoustic displays in TF32 (Milenkovic, 2001). AACT allowed convenient determination of utterance boundaries along with coding in AU, VID, or AV.

In the prior study, infant protophones, cries, and laughs had been located exhaustively throughout each of the 20-min recording sessions using a breath-group criterion (Lynch et al., 1995). Listening supplemented by visual inspection of the high resolution TF32 waveform and spectrogram were used for determining utterance boundaries. This prior coding had involved multiple passes and multiple coders who reached a consensus on utterance locations (for details of the procedure refer the prior study). Utterances of very low intensity (scarcely audible, low perceptual salience) or very short duration (< 50 ms) had not been coded. The decision to leave out such utterances was based on the assumption that utterances of such low perceptual salience would not be likely to have impact upon vocal interaction. This prior coding determined the time frames for judgments made by coders in the present study.

The coders in the present study accessed the previously designated utterances one by one. During AU coding, the video was not shown, and likewise during VID coding, the audio was off, and the acoustic display of the audio signal was not seen. Both modalities were presented simultaneously during AV coding.

### Tasks

The tasks for the coders in the present study were to judge infant affect independently in three conditions—AU, VID, and AV. The coding of infant affect involved a forced choice as either positive, negative, neutral or “can’t see.” The last category was assigned in cases where coders could not see the infant’s face in either of the two camera views. Across the 27 passes of coding (nine infants × three coding conditions), 4 to 24% of the data were dropped due to a report of “can’t see” by at least one coder in either VID or AV (the “can’t see” category was not used in AU). The total number for any affect analysis reported above included 1,019 protophones (Table 1, column 3 SUM), the number of protophones where no coder indicated “can’t see” in any condition. The total number of items for vocal type analysis as indicated in Table 1 included all the protophones coded by both of the coders in each modality with a low of 878 protophones for VID, because cases of “can’t see” prevented any possible coding. Table 1 lists the number of protophones used in every analysis, which depended on the particular analysis type and the number of “can’t sees” occurring in the particular conditions.

The coding of vocalization type was also a forced choice as cry, laugh, squeal, vocant, growl, or silence. This last category was included in order to ascertain whether coders in VID could detect the occurrence of protophones (Hypothesis 9). The last author (who was not a coder for the present study) randomly selected “silence” periods within the silent inter-utterance intervals that would be judged by the coders. The selected silences had durations and SDs of duration comparable to the real utterances of the selected recordings. Prior to coding, the vocal type coders were informed that 10% of the stimuli presented in each coding session would consist of these silences, randomly distributed among the utterance stimuli, and they were informed they could code any interval as “silence” rather than assigning a vocal type during vocal type coding. The goal here was of course to determine whether protophones were detectable in silence by facial movements or postures, and the silence judgments in VID provided a reference point for the protophone judgments—the extent to which listeners would judge protophones as silence or silence as protophones in VID would indicate the extent of failure of VID information in determining the presence of protophone vocalizations. There are no prior empirical data to our knowledge that indicate the extent to which protophones (especially precanonical protophones) involve visible movements of the supraglottal vocal tract or other facial expressions that might indicate vocalization is occurring.

### The Coders and Their Training

Seven graduate students in Communication Sciences and Disorders at the University of Memphis were included as coders, two of them for both vocal type and affect (the first and second authors, a bilingual English-Mandarin speaker and monolingual native English speaker), and the other five (all monolingual native English speakers) for affect only. Training included a single orientation session with the fourth author (a phonetician and a native speaker of English, also competent in Spanish, French, and German), followed by supervised practice sessions over a period of 2 days. The rationale for this brief training is based on the assumption that the affect and vocal categories are natural and universal and that the only requirements of training are to ensure understanding of the category names and to instill confidence in the observers about their intuitive judgments. Coders were encouraged to consider any aspect of audio or video that they felt should contribute to their judgment of affective valence or vocalization type.

The rationale for including more coders on affect than on vocal type was that the vocal type coding, conducted by two coders, yielded relatively unambiguous outcomes with respect to the contributions of the modalities (VID transmitted vocal type poorly, while AU and AV transmitted vocal type well in all analyses). However, for affect coding, preliminary data showed more nuance, with complex variations depending on modality and type of analysis, and so we decided to involve a larger number of coders on affect to increase analysis power.

### Agreement of Current Coders with Coding from the Prior Study

None of the multiple experienced coders that had produced the consensus coding for the prior study (Oller et al., 2013) were among the seven coders for the present study. Still we can compare coding agreement for a small subset of data (not a part of the present set) where both the prior coders and the current ones coded in the same modality for the same set of data. The seven current coders achieved a mean of 0.75 kappa agreement in VID for affect with respect to the prior coding. The two vocal type coders showed a mean of 0.64 kappa agreement in AU with respect to the prior coding. The higher agreement for affect does not appear to be attributable to the larger number of coders. The two vocal type coders had a mean of 0.79 kappa agreement with respect to the prior affect coding.

### Procedure

Overall, the seven affect coders independently completed 27 affect coding passes, and the two vocal type coders completed 27 vocal type coding passes for a total of 243 sessions of coding. The 27 passes were presented in semirandom order for each coder to ensure that coding of any individual session in any of the three conditions would be non-consecutive; by this means we tried to limit the possibility that coders might remember how they had coded individual utterances previously. Thus, for example, a session presented to a coder in VID could not be followed by the same session in AV until at least four other sessions from other infants had been presented to that coder in between. Each of the coders worked with a unique randomized order. For the two coders who worked in both affect and vocal type coding, the two types of task were conducted at different times, with at least 2 months in between, affect first.

### Coder Agreement Measures and Data Analysis (Justification for Using Coder Agreement As the Indicator of Reliability of Signal Transmission in Section S1C in Supplementary Material)

Statistical plan: Our agreement analysis required two input series in which each data point from one series could be paired with a point from the other. Our interest in this case was in the convergence of affect observations and vocal type observations across the coders. We used coder agreement within modalities to measure how reliably AU, VID, and AV transmit information. High agreement in any modality was thus interpreted to suggest the modality carries dependable information.

Cohen’s kappa (Cohen, 1960) was used to assess coder agreement. Unlike percentage of agreement, kappa takes into account the agreement that would be expected purely by chance, indicating the proportion of agreement beyond that expected by chance. In this study, kappa was used to correct for the imbalance of categories—vocants occurred much more often than the other protophones, and neutral affect occurred much more often than the other affect types. We compared each kappa value with chance to assess statistical reliability of coder agreement levels and then compared kappa statistics across conditions for most of the hypotheses using confidence intervals (CI). We did not correct for multiple comparisons across kappa levels. As will be seen below, a variety of possible CI comparisons associated with our hypotheses yielded *p* < 0.001. In other cases reported *p* values were lower, but in all cases both the CI-based comparisons and the numbers of comparisons of coder pairings^6^ that conformed or did not conform to the predictions are supplied.

Hypotheses 4 and 5 required special treatment, which will be explained below. The expected chance agreement was calculated in a way that the distributions of each judged category were taken into account (Sim and Wright, 2005; Reidsma and Carletta, 2008).

We followed Landis and Koch’s recommendations (Landis and Koch, 1977) to interpret the strength of agreement for the kappa coefficient: 0.0 − 0.20 = slight, 21 − 0.40 = fair, 0.41 − 0.60 = moderate, 0.61 − 0.80 = substantial, and 0.81 − 1 = almost perfect. Kappa measures were computed for all possible pairings of coders. Thus in the affect judgments there were 21 pairings of 7 coders, and in the vocal type judgments was 1 pairing of 2 coders. The means of the kappas on affect over those pairings are reported below.

## RESULTS

For perspective, let us begin by pointing out that cry and laugh showed, as expected, very strong tendencies for cry to be judged across the seven coders predominantly as negative (98%) and laugh predominantly as positive (86%) in all three modalities, with neutral judgments accounting for all the cases not conforming to the expectations. The results reported for all the hypotheses are based on agreement data for protophones only. A tabular summary of the outcomes on the nine hypotheses is provided in the Section S1E in Supplementary Material.

### Results on Affect Hypotheses

Hypothesis 1: Intercoder agreement on affect judged in AU measured by kappa (Figure 1), was statistically significant (see text footnote ^6^) (*p* < 0.001, indicating that 99.9% CIs did not overlap with kappa of 0) for all three affect types, at fair (Landis and Koch, 1977) magnitude for positivity and neutrality, and at substantial magnitude for negativity. Therefore, our expectation that infant vocalizations judged in AU transmit reliable affect content in the first year of life was confirmed. In addition, the expectation that negativity would be particularly well transmitted in AU was confirmed, with significantly higher (*p* < 0.001) coder agreement on negativity than on the other affect types (99.9% CIs for kappa agreement on AU negativity did not overlap the means for AU positivity or AU neutrality), with all 21 coder pairings showing higher agreement on negativity than on either of the other types.
Hypothesis 2: Figure 1 also shows confirmation of the hypothesis that affect associated with protophones as judged in VID would yield high intercoder agreement for all affect types (moderate for neutrality, substantial for positivity and negativity) and better intercoder agreement than in AU. For positivity and neutrality, the advantage of VID over AU was great (*p* < 0.001), with all 21 coder pairings (*p* < 0.001) showing higher kappa in VID. But notably, the significant advantage of VID was less prominent for negativity, with 15 of 21 coder pairings showing higher agreement for VID (*p* < 0.05), and both VID and AU agreement values being in the substantial zone.
Hypothesis 3: Next, we examined whether affect associated with protophones as judged in AV yielded better intercoder agreement than either AU or VID. As represented in Figure 1, we observed that coders did agree better for all types of affect in the AV condition than in AU, but they did not uniformly agree better with each other in AV than in VID. Only for negativity did the AV condition yield highest agreement [with AV > VID for 17 of 21 (*p* < 0.005) and AV > AU for 20 of 21 coder pairs (*p* < 0.001)]. For neutrality, AV did not yield significantly better intercoder agreement than VID, with AV > VID for 6 of 21 (*p* > 0.05) and VID > AV for 14 of 21 (*p* > 0.05). Unexpectedly, VID showed higher agreement than AV for positivity [18 of 21 coder pairs (*p* < 0.005)].

### Results on Affect Concordance Hypotheses

Hypothesis 4: First, we probed the question of concordance between AU and VID judgments by asking across coders how often visually judged and auditorily judged affect conflicted in our sample. We had expected relatively rare conflicts (< 0.10). However, the results (Table 2) show non-concordant judgments across AU and VID occurred in more than a quarter of cases across the six cells. Table 2 shows two conflicting patterns for auditorily judged and visually judged affect, (A) one where AU judgments were not confirmed by VID judgments and (B) one where VID judgments were not confirmed by AU judgments. To exemplify: The lower right cell value of 0.13 represents cases where the majority (at least 4) of the seven coders judged an utterance negative in VID, and where fewer than three coders judged the same utterance as negative in AU. In other words, if an utterance was judged to have negative affect based on the face, the judgment based on the voice did not agree 13% of the time. Similarly, the upper left cell value of 0.23 represents cases where at least four coders judged an utterance positive in AU, while fewer than three coders judged the same utterance as positive in VID. In other words, if an utterance was judged to have positive affect based on the voice, the judgment based on the face did not agree 23% of the time.

The data in Table 2 suggest that conflicts were least frequent for judgments of negativity when the VID judgment was negative (lower right cell = 0.13). In contrast, conflicts were much more common when the AU judgment was negative (lower left = 0.37) or when the VID judgment was positive (upper right = 0.37).

These patterns of AU vs. VID conflict in affect judgment can be considered against the background of judgments for cry and laugh. There was not a single instance of such conflict for cry (of 44) and only four for laugh (of 51). This contrast suggests that while face and voice are bound to particular affect types for cry and laugh (the sounds of human infants that resemble animal calls more than the protophones do), the protophones show a much looser connection, consistent with the assumption that they have functional flexibility, and that they are precursors to speech (for analyses of the data on the issue of functional flexibility, Section S1D in Supplementary Material).

Hypothesis 5: Furthermore, after locating all the cases where AU and VID judgments were non-concordant according to the criterion used for Table 2, we constructed Table 3: the numerators of Table 2 became the denominators of Table 3 in order to test for the proportions of cases where AV judgments agreed with VID or AU. The proportions where AV agreed with VID were dramatically higher than the proportions where AV judgments agreed with AU (Table 3: for positivity, 0.76 > 0.05, for neutrality, 0.68 > 0.10, for negativity, 0.75 > 0.16). Table 3 provides further confirmation for the expectation that the video modality takes precedence in judgment of affect.

### Results on Vocal Type Hypotheses

**FIGURE 2:**
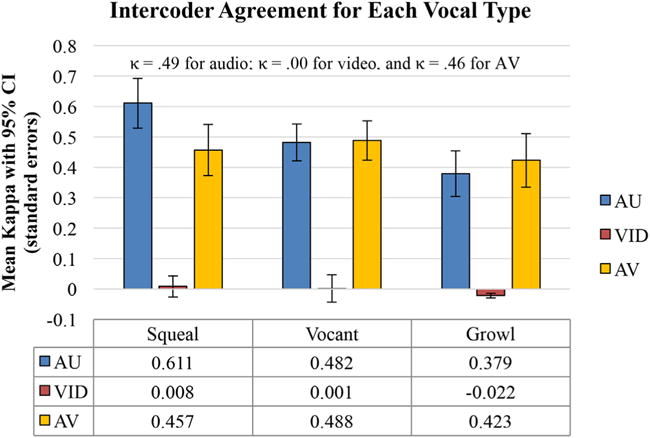
Chance level for these correlations is 0; thus video-only (VID) for squeals, vocants, and growls was not above chance level. The very low correlations suggest that VID provided little if any significant information about protophone type. On the other hand, audio-only (AU) and audio-video (AV) both yielded significant information on all three vocal types. 95% confidence intervals are included. Kappas at the top of the figure are means.

Hypothesis 6: According to the intercoder agreement on vocal type measured by kappa (see Figure 2), coders did not achieve a better agreement level than chance in VID for any of the three affect types. Hypothesis 6 was not confirmed.
Hypothesis 7: Infant vocal types were clearly transmitted better by voice than by face: Intercoder agreement of vocal type judgment in AU was far higher than in VID for all three vocal types (*p* < 0.001), confirming Hypothesis 7 (Figure 2). Squeals showed substantial agreement, vocants showed moderate agreement, and growls showed fair agreement.
Hypothesis 8: Infant vocal types were not found to be better transmitted in AV than in AU, and surprisingly AU actually outperformed AV for identification of squeals (*p* < 0.05). Hypothesis 8 was not confirmed (Figure 2).

### Vocal Type Hypothesis, Detection of Protophones by VID

Hypothesis 9: To probe judgments of protophones by VID further, we sought to ascertain whether coders could discern even the occurrence of protophones in VID. A set of silences (approximately 10% per session) had been selected in random interutterance intervals and had been presented for coding, with coders always having the option of coding “silence.” They assigned “silence” at nearly the optimal rate in VID (9% for both coders), and they did show better than chance identification of the silences, confirming Hypothesis 9, but only weakly. Coders were usually wrong in VID coding about vocalization vs. silence since the two coders failed to code an average of 75% of the actual silences as silences (75% false negatives), and since an average of 77% of their silence designations were mistakes (77% false positives) (Table 4). Coder performance in detecting the silent periods in VID suggested only slight levels of accuracy (Landis and Koch, 1977) [κ = 0.15, 95% CI (0.07, 0.22) for coder 1, κ = 0.19, 95% CI (0.12, 0.25) for coder 2]. In contrast to the very difficult protophone detection in VID, detection in AU and AV yielded kappa values that were almost perfect (κ = 0.92 and κ = 0.95 for AU, κ = 0.93 and κ = 0.96 for AV for the two coders, respectively).

## DISCUSSION

The fundamental question that drives our research is “how did language come to be?” The present work is directed to a specific question about the origin of language: How do the voice and the face play separate and/or coordinated roles in the beginnings of communicative expressions that are precursors to language. The relative roles of face and voice have never been previously evaluated in the first year of human life (in fact never in infancy or childhood) in such a way that the roles can be evaluated both separately and jointly. For this reason we studied coder agreement for AU, VID, and AV judgments of both affect and vocal type.

While facial expressions provided the more reliable basis for affect judgment than vocal expressions, as indicated by intercoder agreement (Figure 1), the data also showed that some affect information was indeed reliably transmitted by the voice as well, as confirmed by the significant values of intercoder kappa agreement for AU (Figure 1). Thus the results show that both the infant voice and the infant face can express affect in protophones.

The results also indicate that the face provides the predominant basis for judgment of affect for protophones, as reflected not just in better intercoder agreement (a predominance that has also been found for nonsense sequences produced by adult actors with differing vocal and facial affect, see Bänziger et al., 2009), but also in how conflicts of affect judgment for the two individual modalities were resolved in the AV condition. For utterances that were judged to have one kind of affect in AU or VID, but not judged to have that type of affect in the other condition, AV judgments conformed overwhelming to the VID judgments (Table 3). The results once again suggest that in language emergence, the face plays a primary role in affect transmission, whereas the voice is partially decoupled from affect, opening the door to the possibility later in life of using the voice in abstract, arbitrary symbol formation.

It is hard to imagine how infants could begin to learn any of the world’s languages in the absence of the ability to use vocal categories flexibly, since this ability underlies any kind of arbitrary vocal symbol learning. Thus, the foundations for speech implied by the early human patterns of vocal expression reported here and in our prior article (Oller et al., 2013) suggest cross-cultural universality of the basic tendencies to use the face and voice in expression of affect and vocal type. At the same time, later development produces many variations in expressive abilities, and thus it should be no surprise if cultures differ to some extent in how they implement vocal and facial expressions in adulthood.

Results both on adult perception of affect as produced by adult actors (Bänziger et al., 2009) and results and reasoning based on research on infant perception of affect (Flom and Bahrick, 2007) suggest that multimodal stimuli (AV) should be more reliably judged than unimodal stimuli (VID). Naturally occurring affect signals are multimodal, and it has been argued the “intersensory redundancy” of such signals facilitates communication and is “no extravagance of nature” (Bahrick et al., 2004, p. 99). However, intercoder agreement in our study for infant affect types judged in AV was not unambiguously better than in VID (see Figure 1).

We are not sure why AV was not uniformly the best condition for agreement on affect, but we have some suggestions about interpretation of the complex results in Figure 1. First, consider the much better coder agreement for affect judgment in AU for negativity than for positivity or neutrality. Perhaps judgments in audio and video together profit from the audio in the case of negativity, but do not profit in the cases of positivity or neutrality, because audio’s potential contribution in those cases is much weaker. In fact the results suggest that the addition of audio to video may actually significantly inhibit identification of positivity.

Another possibility to explain the lack of general advantage of AV invokes the fact that our study focuses on perception of infant, not adult, expressions. Since the actors in the adult study cited above were told what affect to portray for each utterance, it was possible for them to coordinate face and voice to produce unambiguous affect. The babies on the other hand had no instructions and may have often presented ambiguous affect, mixing different vocal and facial affect features. Yet another possibility is purely developmental—that babies in general may not be as good at coordinating face and voice as adults are.

The especially good performance of coders for negativity in AU suggests that the infant voice may be especially adapted for attracting attention when the infant is in distress but not in sight of the caregiver. The acoustics of protophones expressing distress may make them so salient that the sounds alone provide sufficient evidence of infant distress so that caregivers can respond quickly (patterns of caregiver response seen to distress calls of young monkeys, see Owren and Rendall, 2001 and human infant crying, see Papoušek and Papoušek, 1990). The voice appears to transmit urgency especially effectively, and to be well-adapted to request attention or to complain. The data suggest the infant face is a little better than the voice in transmitting negativity (although the caregiver has to be looking at the infant), so we reason that once attention from the receiver is on the infant face, the infant voice is not so important in transmitting affect, because the face can take over, and will in any case do better in transmitting positivity and neutrality than the voice.

The data show that although video was the more reliable modality for affect judgment in protophones, video provided essentially no useful information regarding phonatory vocal type. This finding seems to counter the suggestion that vocalization requires facial movement in primates (Ghazanfar, 2010). The kappa for identification of the three protophone types in the human infants (Figure 2) was nearly 0, and coders identified whether vocalizations occurred or not (through silence recognition) based on VID at only a slight level (Table 4), with both false negative and false positive identifications of silences by video outnumbering hits by nearly 3 to 1.

In our prior article (Oller et al., 2013), it was shown that affect judgments in video corresponded crisply with both illocutionary valence (negativity was systematically interpreted as “complaint or plea,” positivity as “continuation of conversational interaction”) and perlocutionary valence (negativity was responded to by parents with an attempt to change the situation for the baby, positivity with encouragement to continue the conversational interaction). In evolutionary terms, affect can be viewed as a type of expression that influences illocutionary clarity and perlocutionary consistency. The perlocutionary responses of parents can be seen as providing selection pressure on the infant communicative system, and perceived affect seems to heavily drive the decision making of parents regarding their perlocutionary responses (Oller et al., 2016).

The present work has not involved an attempt to evaluate *how* the infant voice simultaneously transmits vocal type information along with affect information in AU. The protophones themselves are known to be differentiable (as vocal types) by pitch and vocal quality parameters (Buder et al., 2008). But there is every reason to suspect that the same parameters are involved in vocal affect (Banse and Scherer, 1996). Other prosodic features are also likely interwoven in both infant vocal type and vocal affect, e.g., variations in loudness, pitch contour, and relative spectral entropy. We do plan research to unravel the complexity of affect transmission by the infant voice, but it is expected to be a challenging task, beyond the scope of the present effort. Also beyond the scope of the present work is any attempt to conduct analyses using automated recognition of facial and vocal cues in infants, an area about which we are enthusiastic for the future.

In conclusion, this work provides documentation in very early human development of a special role for the voice, a role that has not yet been shown in non-human primates. The human infant voice is shown here to be useful in transmission of affect as well as vocal type, while the face is seemingly confined in infancy to expression of affect. The vocal flexibility hints at the possibility that in the evolution of the hominin line, freeing of the voice from obligatory affect transmission may have been a critical step in breaking away from the primate background where voice and face are much more tightly bound in communication.

## Supplementary Material



## Figures and Tables

**TABLE 1:** Nine recordings from nine infants spanning the first year were drawn from a larger study (Oller et al., 2013).

		Affect analyses		Vocal type analyses					
		For all conditions: AU, VID, and AV		AU		VID		AV	
			
Infant	Ages (in months, weeks)	(A) Number of protophones	(B) Number of cries and laughs	(A)	(B)	(A)	(B)	(A)	(B)
1	3, 1	175	7	186	4	133	18	184	12
2	3, 3	99	22	81	21	40	1	75	22
3	4, 1	115	20	114	10	116	7	106	15
4	7, 0	135	0	206	4	124	1	209	4
5	7, 1	103	6	122	10	89	4	111	31
6	10, 1	139	0	179	2	141	0	186	0
7	10, 2	66	10	58	14	61	10	61	14
8	11, 1	111	1	156	12	82	4	152	8
9	11, 2	76	29	135	4	92	0	130	5

Sum		1,019	95	1,237	81	878	45	1,214	111
Mean utterances/session		113.2	10.6	137.4	9	97.6	5	134.9	12.3
SD		37.1	10.7	49.3	6.2	33.9	5.9	51.6	9.7

Over a thousand utterances were coded for affect by seven coders in three modalities (AU, VID, and AV) and for vocal type in the same three modalities by two coders. As in the prior study the number of cry and laugh utterances was, according to the coding, low compared to the number of protophones (<10%).

**TABLE 2:** A presentation of proportions of cases where facial and vocal affect were not judged concordantly in AU and VID for each affect type.

	(A) denominator = # of utterances judged to have the specified affect in AU	(B) denominator = # of utterances judged to have the specified affect in VID

	Numerator = # of utterances where the VID judgment was disconcordant with the AU judgment	Numerator = # of utterances where the AU judgment was disconcordant with the VID judgment
Positive	0.23, 61/262	0.37, 136/369
Neutral	0.24, 104/428	0.23, 99/441
Negative	0.37, 99/267	0.13, 24/182

(A) Cases where affect was judged in AU as positive, neutral, or negative by the majority (at least four) of the seven coders (represented by the denominator in each of the six cells), whereas affect was not judged in VID concordantly by at least three of the coders (represented by the numerator in each of the six cells). (B) Cases where affect was judged in VID as positive, neutral, or negative by the majority, whereas affect in AU was not judged as positive, neutral, and negative by at least three of the coders. Each cell represents a proportion. In all six cells of the table our expectation was violated: non-concordant judgments of affect from VID and AU were not rare (always > 0.10), but accounted for about a quarter of judgments overall. The sum of the denominators in the table does not reach the total N of 1,019, because there were cases (<10%) where the seven coders did not produce a majority of judgments for any of the affect types (e.g., three positive, two negative, and two neutral).

**TABLE 3:** For cases where AU and VID judgments of affect were non-concordant, the table presents the proportions where (A) AV judgments agreed with AU judgments or (B) with VID judgments for each of the three affect types.

	(A) AV agrees with AU when VID is disconcordant with AU	(B) AV agrees with VID when AU is discordant with VID
Positivity	0.05, 3/61	0.76, 103/136
Neutrality	0.10, 10/104	0.69, 68/99
Negativity	0.16, 16/99	0.75, 18/24

The proportions of AV judgments that agreed with AU were significantly lower (p < 0.001, by chi square) for all affect types than those agreeing with VID, which suggests that if vocal and facial affect judgments conflict, the AV judgments tend strongly to agree with VID. As in other cases, the data suggest facial expression tends to predominate in affect judgments.

**TABLE 4:** (A) Data for coder 1 and (B) data for coder 2.

		Silent	Not silent
(A) Coder 1	Silent	24	91
	Not silent	79	1,050
(B) Coder 2	Silent	29	87
	Not silent	81	1,143

When coders used VID to try to detect silent periods (as distinct from protophones), the task was very difficult. False positives (upper right cells for both coders) and misses (lower left cells) substantially outnumbered hits (true positives, upper left cells). Still, observed hits and correct rejections (true negatives, lower right cells) were higher than expected by chance. Kappa after correcting for chance was slight: 0.15 for coder 1, with 95% CI [0.07, 0.22] and 0.18 for coder 2, with 95% CI [0.11, 0.27].

## Footnotes

1 Illocutionary function refers to the communicative force of an utterance, and perlocutionary function to the response that the receiver produces as a result of interpreting the utterance (Section S1B in Supplementary Material for explanations of the use of these terms and their relation with affect).

2 A recent report (Clay et al., 2015) based on observations of wild bonobos has attempted to advance our understanding of functional flexibility in our closest relatives by illustrating *contextual* flexibility in the use of a single call type observed to occur while bonobos were in contexts of feeding, traveling, or engaged in an event of aggression. In fact, the existence of contextual flexibility in primate communication has been reported in a variety of prior studies based on vocal, facial, and gestural actions from non-human primates (Liebal et al., 2013). But neither the new report by Clay et al. nor any prior study has provided clear indications of “functional” flexibility based on variations in transmission of affect, illocutionary force, and perlocutionary force with individual primate calls. We are currently researching this topic with captive bonobos. A controversy exists about referential as opposed to affective communication in non-human primates. One camp argues for referential communication (implying substantial communicative flexibility) (Seyfarth and Cheney, 1986), while another argues that calls are driven by affect and arousal alone (implying much more limited flexibility) (Owren and Rendall, 2001). Recent coverage of the controversy can be found in a volume edited by Stegmann (2013) and in several articles (Fischer, 2013; Rendall and Owren, 2013; Scarantino, 2013; Oller and Griebel, 2014).

3 More recent work from our group showed that flexible relations of protophones with neutral and negative facial affect also occurred in infants from 0 to 3 months of age (positive facial expressions were rare in these very young infants) (Jhang and Oller, 2017).

4 We did not find an age effect in the key measure of intercoder kappa agreement for affect judgments in the three conditions (audio-only, video-only, and audio-video). In addition, we broke the data down into three age groups (early age group, mean = 3.5 months, 371 protophones; middle age group = 7 months, 255 protophones; late age group = 10.8 months, 253 protophones) and found a very similar coder agreement pattern for each of the three age groups and for the total dataset as in Figure 1.

5 The nine selected sessions included naturalistic “interaction” between the infant and the mother as well as periods of infant play “separated” from the mother in the same room while the mother interacted with an experimenter. Eight of the nine sessions contained both mother–infant interaction and separation (or infant play), and one contained only mother–infant interaction. Although face-to-face interaction, as has been required by recording protocols in many prior studies of infancy (e.g., Hsu et al., 2001; Jaffe et al., 2001; Gratier et al., 2015), did occur sometimes in these recordings, the interactions in the present study were not constrained, and there was never any face-to-face requirement. Some infant utterances occurred in interaction from across the room, some in face-to-face interaction, but many utterances occurred with no evidence of interactivity at all. For example, a great many infant utterances occurred when the infant was not engaged in interaction, without gaze directed to any other speaker. These nine sessions are a subset of data from the original PNAS paper (Oller et al., 2013), which included an analysis demonstrating that the distribution of affect with regard to vocal type was similar across both types of sessions (Supporting Figure 12 of the PNAS paper). The data show clearly that both conditions during the recordings provided appropriate data for the analyses on agreement among and within coders.

6 Intercoder agreement values on affect, means and confidence intervals were determined based on kappas computed over 21 coder pairings for each condition (AU, VID, AV) or affect type (positive, neutral, negative). The SEMs for these kappas were multiplied by 1.96 to determine CIs.
