# Supplementary material for: Differing Roles of the Face and Voice in Early Human Communication: Roots of Language in Multimodal Expression
Source: Front Commun (Lausanne). Author manuscript; Available in PMC 2018 Mar 15. (PMC5798486; doi:10.3389/fcomm.2017.00010)
Supplement: Supplementary file 1 [file NIHMS911985-supplement-supplement_1.pdf]

Supplementary material for

Differing roles of the face and voice in early human communication: Roots of language in multimodal expression

Yuna Jhang[1], Beau Franklin[2], Heather L. Ramsdell-Hudock[3], D. Kimbrough Oller[1,4,5]
[1]School of Communication Sciences and Disorders, The University of Memphis
[2]The Institute for Research and Rehabilitation, Memorial Hermann Healthcare
[3]Department of Communication Sciences and Disorders, Idaho State University
[4]Konrad Lorenz Institute for Evolution and Cognition Research, Klosterneuburg, Austria
[5]Institute for Intelligent Systems, The University of Memphis

Section A. Precanonical protophones of infancy and canonical babbling

Protophones are those vocalizations of infants that are the presumed precursors to speech. We draw the distinction between the early developing precanonical protophones, which do not require any supraglottal articulation of the vocal tract, and the canonical protophones that begin to appear in the second half year, that include single well-formed syllables such as [ta] or [na] and highly salient reduplicated canonical syllable sequences such as baba, dada, yaya…. Protophones are not vegetative sounds (such as coughs, sneezes, hiccoughs. burps…), which are presumed to have communicative import only incidentally, since their primary functions are physiological (clearing the airway, managing certain digestive functions…). They are also not "fixed signals" (Lorenz, 1951), such as cry or laughter, sounds that are assumed to be naturally selected as communicative signals, although these tend to have predeterrmined functional valence (negative for cry, positive for laugh) in infancy. All the protophones, in contrast to fixed signals, are flexibly associable with any functional/affective valence (Oller et al., 2013).

Some of the most prominent early infant vocalizations are defined exclusively by the type of phonation (i.e., the typical source in the source-filter theory of speech production; cf. Fant,

1960) that characterizes them, and the present study focuses in its coding on three types of phonation typically used in infant vocalization (Buder et al., 2008). Vocants (or vowel-like sounds), for example, are characterized by normal phonation and are produced in the standard pitch range of the speaker's voice (i.e. modal). Squeals, on the other hand, are characterized by high pitch and typically by a phonatory pattern called loft or falsetto. Growls are characterized by any of several phonatory patterns that include notable aperiodicity (subharmonics, biphonation, chaos..), resulting in rough sounding voice. When these phonatory patterns are produced in the absence of any supraglottal articulation (and they often are, especially in the first half year of life), they are precanonical, and are categorized simply as vocants, squeals, or growls.

Of course all these phonatory patterns can, and regularly do, also occur during more advanced vocalizations. Thus a single canonical syllable can occur in any of the three phonatory patterns, in which case it is categorized phonatorily as a vocant, squeal, or growl, while also being categorized in terms of supraglottal articulation (the filter in Fant's source-filter theory) as having all the features of canonical babbling (including a consonantal margin and a well-formed transition from margin to nucleus [the vowel-like portion of the utterance]). This single canonical syllable can also be characterized in terms of its particular types of manner or place of articulation of the supraglottal tract.

The three phonatory patterns we focus on in this study can be (and are) utilized throughout life as registers of speech—thus adults can squeal as they produce any sentence, and often do when talking to babies. Imagine producing "Aren't you cute?" in infant-directed speech, with very high pitch, falsetto voice, and a wide pitch range. Such an utterance is a squeal, while also constituting a well-formed mature sentence.

The coding procedure in the present work was designed to focus on the phonatory categorizations only, but still, it should be clear from the foregoing definitions, that these categorizations apply equally to precanonical and canonical vocal types, both of which occurred in the samples that were coded. The coding did not include indications discriminating between canonical and precanonical protophones, but the criteria for assignment to the phonatory categories were identical. Consequently, some of the utterances evaluated, especially at the older ages (≥ 7 months old), *did* include canonical syllables categorized as squeal, vocant, or growl, because they possessed one of the three phonatory characteristics corresponding to those categories.

Section B. Affect as a determiner of communicative function in infancy: Illocutionary and perlocutionary forces

Of course transmission of affective states is only one aspect of communicative function in adult language, but in the case of communication in the first year, affect is central to communicative function. Immediate functions of communication by both adults and infants can be portrayed as "illocutionary forces" in the terminology of Austin (1962). In our usage of the term, "illocutionary forces" are sometimes initiated by nothing more than the *expression of an emotional state* but at other times (especially in adults), they involve a communicative intention on the part of the sender, such as complaint or refusal, which may or may not be accompanied by a clear expression of emotional state. A second function of communication pertains to the response that occurs in the receiver as a result of interpreting the sender's communication (including its illocutionary force, whether intentional or unintentional). The response of the receiver, also in our adaptation of Austin's terminology, is the "perlocutionary effect". Austin's terms have been adapted and extended for use in studies of development and cross-species

comparisons (Oller, 2000; Oller & Griebel, 2008; Oller, Griebel, & Warlaumont, 2016). In accord with this theoretical view of communicative functions in infancy, perlocutionary force is a change of state or an action by caregiver/receivers (occurring after having interpreted the sender's communication), which feeds back in the form of selective pressure on potential future communications by infant/senders (Oller, Griebel, & Warlaumont, 2016).

Our research focuses on infant affect transmission during vocalization because affect naturally constrains the range of illocutionary and perlocutionary forces that are possible in infant vocal communication to certain valence classes (positive, neutral, or negative). Positive affect during vocalization can be interpreted by caregiver/receivers as exultation, encouragement to continue interaction, and so on, all of which are naturally *positive* illocutions (Oller et al., 2013). By contrast, negative affect can be interpreted by caregiver/receivers as rejection, complaint, or mere distress expression, all of which are naturally *negative* illocutions. In accord with the valence constraint, positive illocutions are constrained *to remain within their valence class* by their affect, and as a result, positive affect during an infant vocalization cannot, for example, be interpreted as complaint. Thus affect transmission (even transmission of neutral affect) is a key beginning point in the *functions* of communicative acts (Oller et al., 2013). For these reasons, it is sensible, we believe to address the infant earliest communicative functions by grouping them into the three valence classes (positive, neutral, negative) on the basis of affect.

Section C. Coder agreement as the optimal indicator of reliability of signal transmission in infant vocalizations and affect

As indicated in the main text, we reason that intercoder agreement provides the optimal measure of reliability of transmission of vocal type and affect in infancy. In addition to the reasons presented for this viewpoint in the main text of the article, the rationale for this thinking

is based on the fact that infants receive care on the basis of their perceived state. Consequently the states transmitted by infants must be under positive selection pressure (both in development of the individual infant and in the evolution of signals in the species). If the signals could not be consistently recognized by potential caregivers, they would surely fail to elicit care as needed and be eliminated from the pool of behaviors of infants—it is reasonable to assume they would be replaced by signals that *did* elicit care as needed. Otherwise the signal system would collapse (Maynard & Harper, 2003). Consistency of recognition implies that a variety of potential caregivers should agree on signal type, and lack of agreement implies inconsistency of transmission of the signal. Our paper thus assesses the relative consistency of transmission in infancy of affect and vocal type by face, voice, and their combination by using the proxy of coder agreement. Coder agreement among adult observers, all of whom are potential caregivers of infants, appears to us to provide the ideal measure of this consistency.

We might ask what other alternative exists? We can imagine one. For example, we might propose automated measures of any of these variables. However, the automation proposal implies an evaluation procedure, and indeed the quality of the automated measures would have to be judged against their agreement with some human observer or pool of human observers, because the point of the automated measure would be to simulate human judgment. Furthermore, no practical automated measures of affect and vocal type for infants are yet available that could have tested the questions we addressed.

As cited in our paper, we are not the first to pursue assessment of signal transmission using coder agreement as the measure. For example, Green et al. (1995, as cited in the main text) measured differentiation of cry and non-cry vocalizations in infants in different modalities, by assessing agreement between one set of human observers (a presumed gold standard) and a pool

of additional observers. They concluded that recognition of cry vs. non-cry was indicated by the level of agreement. We see no reasonable alternative available to an approach comparing observer agreement. Unlike Green et al., we have assumed no gold standard, because we see no clear basis for determining a gold standard—any human listener is a potential caregiver and thus must be capable of judging infant affect and vocalizations. We might ask, who among us could be determined objectively to be the best judge of infant affect or vocal type? Since we have no good empirically-based answer to the question, we conclude coder agreement is a reasonable measure on its own.

We do not seek to determine the "true affect" or the "true vocal types" transmitted by infants with each communicative event, since again, we have no basis for determining a single gold standard. Instead we choose to have multiple adult observers judge these phenomena with equal opportunity in the three modalities. In this way, the degree of observer agreement can be taken reasonably as an indicator of the degree to which the signal (affect or vocal type) in each modality has been naturally selected to be recognizable as an affect or a vocal type signal.

Section D. Analyses of the Data on the Issue of Functional Flexibility

Functional flexibility revisited

In order to ensure that the 9 recordings selected for the present study were representative in terms of the principle of functional flexibility in infant protophones to the broader sample of recordings from which they were drawn, we conducted analyses perfectly parallel to those of the prior study that had used the full set of 54 recording (Oller et al., 2013). In addition, we were able to analyze the data to address two issues that have not been previously studied.

The first of these analyses (See below, SI Table 1) was identical to that of the prior study (the one involving video-only affect judgments by the coders), while the other two were parallel,

but new (the ones involving audio-only and audio-video judgments). The first question we addressed, then, was whether the functional flexibility pattern would be manifest in the smaller sample (as it had been in the larger one) for *video-only* judgments? The second question addressed an issue that as far as we know has never been previously evaluated, namely, whether the functional flexibility pattern would be manifest for *audio-only* judgments. A positive finding would suggest that there is significant information about affect in infant protophones *as sounds*, independent of their possibly accompanying facial expressions. A positive finding would also suggest that infant control of each of the three types of phonation is not bound to a particular affect valence, but that all three types can transmit all three valences by acoustic means. For the third question, we saw no reason to expect that using *audio-video* stimulus presentation would yield a different pattern of results with regard to functional flexibility than video-only, since we expected facial affect information to play a strong role in overall interpretation of affect whether or not audio was also used for the judgments.

SI: Table 1. Functional Flexibility of protophones for three conditions

	**Questions**	**Analysis approach**
Q1	Functional (affect) flexibility occurs in protophones in video-only	Odds ratios
Q2	Functional (affect) flexibility occurs in protophones in audio-only	Odds ratios
Q3	Functional (affect) flexibility occurs in protophones in audio-video	Odds ratios

To ensure that the subset of data we selected (9 infant sessions) were on the whole representative of the larger set of 54 sessions used in the prior functional flexibility study, we explored the dataset first in terms of similarity on numbers of utterances and vocal types. 1/6 of the 54 recorded sessions from the larger sample had been selected, and indeed the number of

utterances in the selection was near 1/6 of the total for the larger sample. In addition the percentage of cry and laugh to all utterances was <10% in both the selected sample of 9 recordings and in the whole dataset of 54 recordings.

More importantly, we considered the six predictions of the prior study regarding functional flexibility using odds ratio (OR) tests, as had been done previously. For the present study the six predictions focused on how affect was perceived in the three conditions, and enabled us to compare the current results with the prior results on the larger sample: Protophones were expected to show 1) more affective positivity than cries, 2) less positivity than laughs, 3) more neutrality than cries, 4) more neutrality than laughs, 5) less negativity than cries, but 6) more negativity than laughs (see Table 2). In the prior study these predictions were evaluated exclusively with video-only judgments of affect.

Q1: The six patterns in the current data were very similar to those reported in the prior study, using the very same procedure of *video-only* affect judgment, even though the sample size was much smaller (SI: Fig 1A and SI: Table 2). Thus even in this smaller sample, there was a dramatic contrast in flexibility of usage between the protophones (the presumed precursors to speech) and cry/laugh. Cry and laugh are often analogized to animal calls, which are generally treated as more bound to particular states than protophones are, although research suggests notable context flexibility and gradedness of animal call (Hopkins, Taglialatela, & Leavens, 2011; Slocombe & Zuberbühler, 2007).

SI: Table 2. Odds Ratios across protophone types and conditions.

	Video-only affect judgments			Audio-only affect judgments			Audio-video affect judgments		
	SQ	VOC	GR	SQ	VOC	GR	SQ	VOC	GR
**Protophone > cry in positivity**	59.2*	45.9*	59.2*	180.3*	122.9*	83.3*	234.9*	340.8*	415.8*
**Protophone < laugh in positivity**	20*	13.5*	10.5*	4.3*	6.3*	9.3*	26.7*	18.4*	15.1*
**Protophone > laugh in neutrality**	2.7*	7.5*	6.2*	0.4[1]	3.6*	3.9*	3.0*	9.8*	8.4*
**Protophone > cry in neutrality**	6.7*	18.5*	15.2*	1.1[2]	10.1*	10.9*	10.6*	34.6*	29.4*
**Protophone < cry in negativity**	18.4*	81.1*	88.9*	9.5*	33.2*	25.7*	38.7*	174*	182*
**Protophone > laugh in negativity**	82.3*	18.6*	17.0*	12.2*	3.5*	4.5*	78.3*	17.4*	16.6*

54 Odds Ratios are presented for six predictions regarding functional flexibility in accord with the prior study (Oller et al., 2013). The cell values are the odds obtained for each prediction. For instance, squeals were 180.3 times more likely to occur with positive affect than cries when affect was judged in audio-only (fourth column, top row). Notice that for all but two predictions, the odds ratios statistically supported the patterns reported in the prior study (* represents $p < .0001$), even though the dataset here for each prediction was only about 1/6 the size of that in the prior study.

[1]The value .4 (being lower than 1, column 4, row 3) indicates that squeals were significantly *less* neutral than laughs in audio-only.

[2]The value 1.1 is very near chance and not statistically significant.

Q2: The pattern for *audio-only* judgments of affect (SI: Fig 1B) was also similar to that of the prior study, showing for the first time to our knowledge, that functional flexibility of infant sounds can be discerned by voice alone. But still the odds ratios tended to be lower in the audio-only condition than in the others (video-only and audio-video), suggesting weaker conformity to the six predictions. All the ORs in the present study for video-only and audio-video showed significant ORs, and 16 of the 18 predictions for audio-only showed significant ORs, again

confirming the dramatic difference in flexibility between the protophones and cry/laugh. Notice that the smallest OR obtained for Table 3 concerned audio-only judgments of affect in the comparison of squeals and laughs for neutrality (OR = .4). Indeed, the number <1, indicates that squeals were judged in the audio-only condition to be *less* neutral than laughs (see SI: Fig 1B), conflicting with the results of the prior study. Further, squeals judged in audio-only were not significantly more neutral than cry, as suggested by the OR scarcely higher than 1. But only these two of the 18 predictions for audio-only in SI, Table 2 and reflected in data from SI, Fig 1B failed to significantly conform to the predictions.

Q3: The audio-video pattern of results in SI: Table 2 and Fig 1C shows very strong conformity to the six predictions, indicating functional flexibility of the protophones in contrast to functional rigidity of cry and laugh. The proposition was confirmed, in that audio-video yielded a very similar pattern of results to that of video-only (SI: Fig 1A).

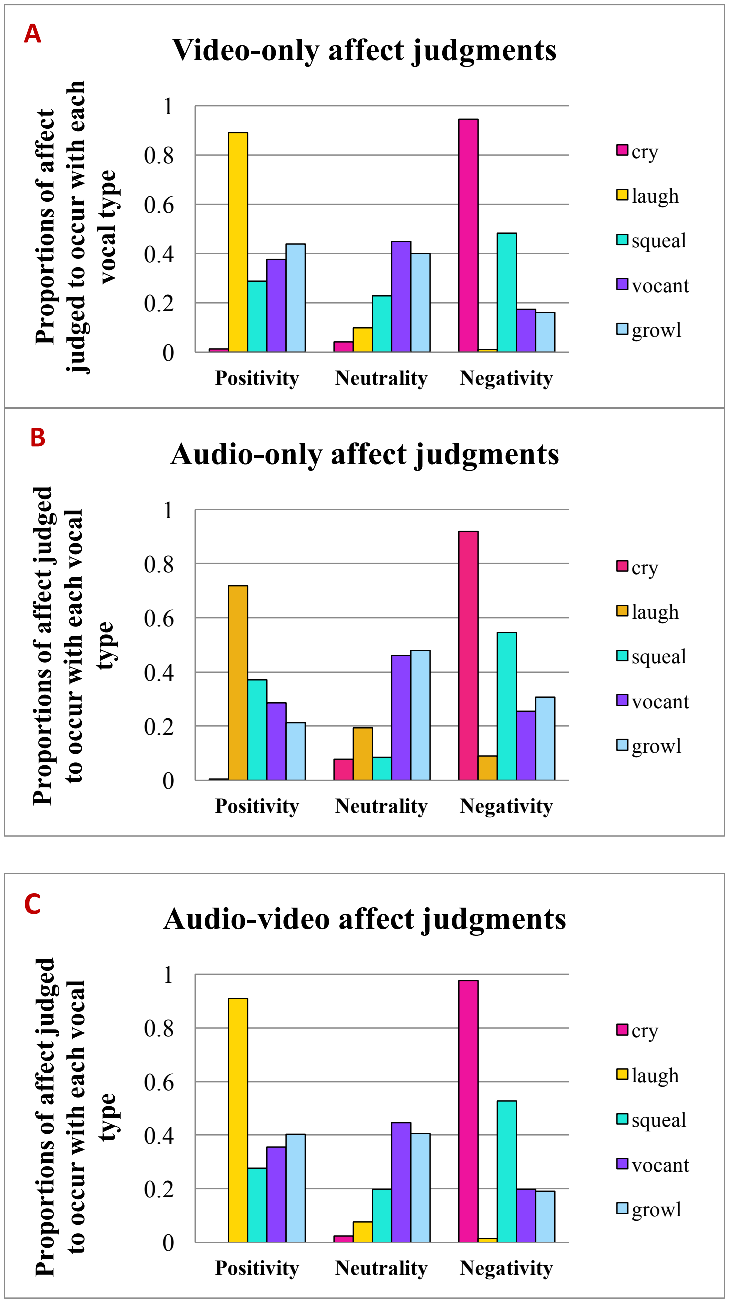

SI: Figure 1. Data on affect judgments in three conditions illustrating conformity of the data to the six predictions of the prior study (Oller et al., 2013) as portrayed in SI: Table 2. In all but two cases out of 54, the predictions of the prior study regarding functional flexibility of the protophones were confirmed. (A) *Data on video-only judgments for 18 predictions*. Here the pattern is nearly identical to that found in the prior study. (B) *Data on audio-only judgments for 18 predictions*. Even in audio-only, judgments of affect basically conformed to patterns of the prior study, suggesting functional flexibility of the protophones in contrast to relative functional invariance of cry and laugh. The infant voice was thus shown to carry significant affect information. (C) *Data on audio-video judgments for 18 predictions*. Strong conformity to the predictions and similarity to the video-only pattern is illustrated.

Section E. Summary of findings

SI: Table 3. Summary of both affect and vocal type propositions and outcomes from the main text.

Hypotheses	Outcome and Supporting Information
1	The infant voice transmits affect in protophones, but most effectively for negative affect: Results reported in Fig 1 confirm the hypothesis
2	The infant face transmits affect more reliably than the voice during protophones: The hypothesis was confirmed by intercoder agreement for affect judged in video-only being statistically reliably higher than in audio-only for all three affect types: Fig 1
3	The infant voice and face together transmit affect most reliably: Not confirmed by kappa analysis: Fig 1
4	The infant face and voice are highly concordant in affect transmission: In fact about a quarter of affect judgments were disconcordant for audio-only and video-only: Table 3
5	The face will predominate in transmission of infant affect: The data showed that indeed if vocal and facial affect judgments conflict, the AV judgments tend strongly to agree with VID: Table 4
6	Infant vocal types (squeal, vocant, growl) will be transmitted significantly better than chance by the face alone: Not confirmed by kappa analysis, Fig 2
7	Infant vocal types will be transmitted better by voice than by face: Confirmed by kappa analysis, Fig 2
8	Infant vocal types will be transmitted better by a combination of face and voice than by voice alone: Not confirmed, Fig 2
9	Infant protophones will be differentiable from silence with facial cues only: Confirmed, but still false positives and misses outnumbered hits for silence judgments by three to one: Table 5

References

Lorenz, K. (1951). Ausdrucksbewegungen höherer Tiere. Naturwissenschaften. 38, 113-6.

Oller, D. K., Buder, E. H., Ramsdell, H. L., Warlaumont, A.S ., Chorna, L., Bakeman, R. (2013). Functional flexibility of infant vocalization and the emergence of language. *Proceedings of the National Academy of Sciences, 110*(16), 6318-632. doi: 10.1073/pnas.1300337110.

Fant, G. (1960). Acoustic theory of speech production. s'Gravenhage: Mouton.

Buder, E. H., Chorna, L., Oller, D. K., Robinson, R. (2008). Vibratory regime classification of infant phonation. *Journal of Voice*. 22, 553-64.Austin, J. L. (1962). How to do things with words. London: Oxford Univ. Press.

Oller, D. K. (2000). The emergence of the speech capacity. Mahwah, NJ: Lawrence Erlbaum Associates.

Oller, D. K., Griebel, U. (2008). Complexity and flexibility in infant vocal development and the earliest steps in the evolution of language. In D. K. Oller & U. Griebel, (Eds.), Evolution of Communicative Flexibility: Complexity, Creativity and Adaptability in Human and Animal Communication (p. 141-68). Cambridge, MA: MIT Press

Oller, D. K., Griebel, U., & Warlaumont, A. S. (2016). Vocal development as a guide to modeling the evolution of language. *Topics in Cognitive Science*, *8*(2), 382–392. http://doi.org/10.1111/tops.12198

Maynard, S. J., Harper, D. (2003). Animal signals. Oxford: Oxford University Press.

Hopkins, W. D., Taglialatela, J., Leavens, D. A. (2011). Do apes have voluntary control of their vocalizations and facial expressions? In A. Vilain, J. L. Schwartz, C. Abry, J. Vauclair, (Eds.), Primate communication and human language (p. 206-26). Amsterdam: John Benjamins Publishing.

Slocombe, K. E., Zuberbühler, K. (2007). Chimpanzees modify recruitment screams as a function of audience composition. *Proceedings of the National Academy of Sciences, 104*(43), 17228-33.
